# Atypical modulation of distant functional connectivity by cognitive state in children with Autism Spectrum Disorders

**DOI:** 10.3389/fnhum.2013.00482

**Published:** 2013-08-27

**Authors:** Xiaozhen You, Megan Norr, Eric Murphy, Emily S. Kuschner, Elgiz Bal, William D. Gaillard, Lauren Kenworthy, Chandan J. Vaidya

**Affiliations:** ^1^Department of Psychology, Georgetown UniversityWashington, DC, USA; ^2^Children's Research Institute, Children's National Medical CenterWashington DC, USA

**Keywords:** fMRI, intrinsic, spontaneous, task, ASD

## Abstract

We examined whether modulation of functional connectivity by cognitive state differed between pre-adolescent children with Autism Spectrum Disorders (ASD) and age and IQ-matched control children. Children underwent functional magnetic resonance imaging (fMRI) during two states, a resting state followed by a sustained attention task. A voxel-wise method was used to characterize functional connectivity at two levels, local (within a voxel's 14 mm neighborhood) and distant (outside of the voxel's 14 mm neighborhood to the rest of the brain) and regions exhibiting Group × State interaction were identified for both types of connectivity maps. Distant functional connectivity of regions in the left frontal lobe (dorsolateral [BA 11, 10]; supplementary motor area extending into dorsal anterior cingulate [BA 32/8]; and premotor [BA 6, 8, 9]), right parietal lobe (paracentral lobule [BA 6]; angular gyrus [BA 39/40]), and left posterior middle temporal cortex (BA 19/39) showed a Group × State interaction such that relative to the resting state, connectivity reduced (i.e., became focal) in control children but increased (i.e., became diffuse) in ASD children during the task state. Higher state-related increase in distant connectivity of left frontal and right angular gyrus predicted worse inattention in ASD children. Two graph theory measures (global efficiency and modularity) were also sensitive to Group × State differences, with the magnitude of state-related change predicting inattention in the ASD children. Our results indicate that as ASD children transition from an unconstrained to a sustained attentional state, functional connectivity of frontal and parietal regions with the rest of the brain becomes more widespread in a manner that may be maladaptive as it was associated with attention problems in everyday life.

## Introduction

Disturbed functional connectivity across distant regions is posited to mediate functional impairment in Autism Spectrum Disorders (ASD). Functional impairment in ASD comprises symptoms of ASD (e.g., difficulty with social interaction and communication, repetitive and restricted behaviors and interests) as well as problems with executive function, the goal-directed regulation of attention, actions and thoughts (Hill, 2004; Kenworthy et al., 2005, 2008). While executive dysfunction is not part of ASD diagnosis, it is associated with symptom presentation (e.g., Lopez et al., 2005; Kenworthy et al., 2009; Yerys et al., 2009a) and decreased independence and poor outcomes in adulthood [see review Hume et al. (2009)]. An emerging theoretical view of ASD is that frontal-posterior temporal synchronization of blood-oxygen level dependent (BOLD) signal is reduced in ASD subjects while they are engaged in social/communicative or executive functions (Just et al., 2012). Such “underconnectivity” has also been observed in spontaneous low-frequency BOLD fluctuations while subjects are not engaged in a directed task, a state of unconstrained cognition that is referred to as “resting” (Cherkassky et al., 2006; Kennedy and Courchesne, 2008; Assaf et al., 2010; Weng et al., 2010; Wiggins et al., 2011; Gotts et al., 2012; Von dem Hagen et al., 2012). In addition to evidence supporting underconnectivity in ASD, greater than normal functional connectivity (“overconnectivity”) has also been noted, either across cortical regions or between subcortical and cortical regions, during task-evoked (Noonan et al., 2009; Shih et al., 2011; Lai et al., 2012) as well as resting (Monk et al., 2009; Di Martino et al., 2011) states. Cognitive conditions that yield abnormally weaker or stronger functional connectivity in ASD are currently not well understood (Müller et al., 2011).

Functional connectivity may be atypical in ASD not only with respect to overall strength but also in its modulation by cognitive state. Studies of healthy adults show that the topology of functional network organization is remarkably similar during task-evoked and resting states. Networks delineated from spontaneous BOLD fluctuations while subjects rest (termed intrinsic connectivity networks) conform to activation patterns observed during visual, auditory, sensorimotor, executive, and self/internally-oriented tasks (Smith et al., 2009) and predict individual differences in task-evoked activation and associated performance (Fox et al., 2006; Mennes et al., 2010; Gordon et al., 2012c). Further, intrinsic connectivity networks are preserved during sleep (Fukunaga et al., 2006) and light anesthesia (Vincent et al., 2007; Greicius et al., 2008), suggesting that they do not depend upon conscious cognition. While their topology is preserved across states, their strength differs in several ways: First, intrinsic connectivity was stronger, within networks and in anticorrelation across networks, during awake than non-conscious states [see review Heine et al. (2012)]. Second, within-subjects' comparison showed that functional connectivity became stronger from resting to a task-evoked state selectively, in regions activated during the task such as auditory (Arfanakis et al., 2000), visual (Arfanakis et al., 2000; Hampson et al., 2004; Nir et al., 2006), or motor (Arfanakis et al., 2000; Jiang et al., 2004). Third, functional connectivity decreased across some networks during task performance relative to a resting state (Fransson, 2006; Gordon et al., 2012b), suggesting that specific networks became more segregated when subjects were in a cognitive state constrained by a task. Fourth, the extent to which functional connectivity changed from resting to task states, particularly across networks, varied across individuals based upon dopamine neurotransmitter function and traits of distractibility and impulsivity (Gordon et al., 2012b). Together, these findings support the notion that functional connectivity is dynamic, and its modulation by cognitive state is associated with individual variability in attentional function. Whether state-related changes in functional connectivity are atypical in ASD and whether they predict attentional function is unknown.

The goal of the present study was to examine whether changes in functional connectivity, from a resting to a sustained attention state differ between ASD and typically developing (control) 9–13 year-old children. We focused on this narrow age range later in childhood in order to minimize developmental differences and maximize chances of acquiring two motion-free back-to-back fMRI runs from each child. We measured the strength of functional connectivity using a voxel-wise method that distinguished local connectivity, defined as within a voxel's 14 mm neighborhood, and distant connectivity, defined as connectivity of a voxel to the rest of the brain, outside of its 14 mm neighborhood. Such a voxel-wise data-driven method allows testing predictions without regard to a priori functional divisions, an approach that distinguishes the present study from past functional connectivity studies of ASD. For distant connectivity, we predicted a Group × State interaction such that control but not ASD children would modulate connectivity in response to the sustained attention state. As adult findings reviewed above showed that connectivity of selective networks became stronger during a task relative to a resting state, we reasoned that in control children, such a change suggestive of focal connectivity networks (i.e., task-relevant connections get stronger while task-irrelevant connections get weaker) ought to be expressed as a net reduction in our estimate of distant connectivity, which considers all voxels in the brain. In contrast, in light of the many underconnectivity findings in ASD during both task-evoked and resting states reviewed above, we expected overall weaker distant connectivity and little change from resting to task states. Further, we also explored whether whole-brain metrics of connectivity using two graph theory measures, global efficiency and modularity, would be sensitive to Group × State interaction. Global efficiency, measured by path length and reflecting network integration, characterizes the average “speed” of information transfer between any pair of nodes (Latora and Marchiori, 2001; Achard and Bullmore, 2007), and was lower in ASD subjects in a resting state magnetoencephalography study (Tsiaras et al., 2011). Modularity, on the other hand, reflects network segregation, through defining how well an entire network is organized into modules of densely interconnected nodes (Newman, 2006), and was higher in ASD subjects in a resting state electroencephalography study (Barttfeld et al., 2011). For regions (and graph theory metrics) showing the predicted interaction, we examined whether the state-related change in functional connectivity was related to attention problems measured by the inattention score of the ADHD Rating Scale (DuPaul et al., 1998). We focused upon attention, rather than hyperactivity/impulsivity or ASD symptoms, as it is most closely related to sustained attention, the task-state examined here. Due to the lack of past work on local connectivity changes by state in healthy or ASD adults or children, we tested for the same Group × State interaction but made no predictions.

## Methods

### Subjects

Thirty-one children aged 9–13 years, 15 with a diagnosis of ASD (3 left handed and 12 right handed) and 16 control children (all right handed), matched for age, IQ, and gender (see Table 1), participated in the study after complying with consenting guidelines of the Georgetown University and Children's National Medical center Institutional Review Boards. This sample was retained after applying criteria for head motion, from a total sample of 24 ASD and 26 control children. ASD children were recruited through the Center for ASD at Children's National Medical Center. Control children were recruited from the Washington DC area community through advertisements at public venues and pediatrician offices.

**Table 1:** **Demographic characteristics (Mean and standard deviation in parenthesis)**.

	**ASD**	**Control**
*N*	15	16
Gender (females) (χ^2^ = 1.06, *p* = 0.30)	3	7
Age (in years) (*p* = 0.96)	11.2 (1.4)	11.2 (1.3)
Full scale IQ (*p* = 0.26)	118.7 (11.5)	123.0 (9.2)
Performance IQ (*p* = 0.17)	112.7 (12.9)	118.5 (9.8)
Verbal IQ (*p* = 0.44)	120.4 (11.3)	123.5 (11.0)
ADHD Rating scale inattentive raw score (0–25) (*p* < 0.0001)	13.9 (6.2)	4.1 (3.5)
ADHD Rating scale hyperactive/impulsive raw score (0–25) (*p* < 0.001)	8.6 (5.5)	2.4 (3.0)
ADOS Communication total (1–7)	3.0 (1.8)	–
ADOS Social interaction total (2–13)	7.5 (3.1)	–
ADOS Stereotypical behaviors and restricted interests total (0–5)	1.8 (1.7)	–
ADI-R Total verbal score (7–24)	16.0 (4.8)	–
ADI-R Total social interaction score (11–28)	19.9 (5.5)	–
ADI-R Restrictive interests and repetitive behaviors score (3–7)	4.9 (1.3)	–

ASD case classification followed diagnosis by a trained and experienced clinician based on the DSM-IV-TR criteria (American Psychiatric Association, 2000) and was confirmed with the Autism Diagnostic Interview—Revised (ADI-R) (Lord et al., 1994) and the Autism Diagnostic Observation Schedule—Generic (ADOS-G) (Lord et al., 2000) following the criteria established by the NICHD/NIDCD Collaborative Programs for Excellence in Autism (Lainhart et al., 2006). These criteria require that the child meet ADI-R cutoff for autism in the social domain and at least one other domain (communication and/or repetitive behaviors and restricted interests), and meet ADOS cutoff (autism or ASD) for the combined social and communication score. One ASD subject met criteria for an ASD diagnosis on the ADI and ADOS, and by clinical diagnosis two years prior to this study, but on re-evaluation showed significant improvement on the ADOS.

Exclusion criteria included: (1) Full-Scale IQ below 80 as measured by the Wechsler Intelligence Scale for Children (WISC-IV) or Wechsler Abbreviated Scale of Intelligence (WASI) (Wechsler, 1999); (2) Other neurological diagnosis(e.g., epilepsy) based on parent report; (3) Psychiatric diagnosis based on Child and Adolescent Symptom Inventory—4R (Lavigne et al., 2009) for control children; and (4) Contraindications for MRI such as metallic implants or pregnancy. We used the WISC-IV General Ability Index (GAI) as a measure of Full Scale IQ. The GAI provides a comparable approximation of overall intellectual ability as represented by the WISC-IV Full-Scale IQ score, yet is less sensitive to the influence of working memory and processing speed (Prifitera et al., 1998; Weiss et al., 1999; Saklofske et al., 2004). For participants with WASI scores, we used the Tellegen and Briggs (1967) formula to convert WASI subtest scores into WISC-IV Index scores. In addition, we collected the ADHD Rating Scale: Home Version from parents (DuPaul et al., 1998). Five children in the ASD group were on stimulants that were withheld for at least 24 h before scanning; in addition one child with ASD was on non-stimulant and anti-anxiety medications that could not be withdrawn. All remaining children were not medicated.

### Imaging protocol

Echo-planar images were acquired on a Siemens Trio 3T with parameters: 3 mm isotropic resolution (3.0 × 3.0 × 2.5 mm), *TR* = 2000 ms, *TE* = 30 ms, flip angle = 90°, FOV = 192 × 192 mm. Each child underwent two functional runs, a resting state run for 5:14 min in which children were asked to rest with eyes open and stay awake, followed by a task run during which children performed a sustained attention task modified from Zink et al. (2003). Children were instructed to focus on the center of the screen and press a button with their right hand for a triangle (target stimuli) among serially presented squares, circles, and rectangles, and to ignore anything else that may come up elsewhere on the screen. Each stimulus was presented for 750 ms within a 2000 ms interstimulus interval. Targets appeared on 25% of the trials and the remaining trials were non-targets, requiring no motor response. Of these non-target trials, 25% were presented with the central stimuli only and on the remaining trials, a distracter, a small flickering shape was flashed in the periphery in one of the four corners of the display. On half of these distracter trials, the flickering shape was an open circle, whereas on the remaining half of the distracter trials, the shape was variable (e.g., star, diamond) and colorful. Therefore, the breakdown of the types of trials was 25% target, 25% non-target without distracter, 25% non-target with familiar distracter, and 25% non-target with novel distracter. The task consisted of 168 total trials presented in an event-related design with appropriate jitter determined by Optseq2 (http://surfer.nmr.mgh.harvard.edu/optseq/) and lasted 5:46 min. Trial types are not pertinent to the present results as they were regressed out from the connectivity analysis, and therefore, the only difference in connectivity between the resting and task runs was driven by the attentional state of the subject, unconstrained in the resting run and sustained in the task run. Structural images were also acquired for each subject, with a high resolution sagittal T1-weighted structural scan using a 3D MPRAGE sequence with a scan time of 8:05 min and the following parameters: *TR* = 2530 ms, *TE* = 3.5 ms, 256 × 256-mm FOV, 176-mm slab with 1-mm-thick slices, and a 7° flip angle. Head motion was minimized by foam cushions padding the space between the subject's head and the headcoil.

### Image preprocessing

Images were processed in SPM8 (Wellcome Department of Cognitive Neurology, London, UK) using MATLAB (Version 7.1 Mathworks, Inc., Sherborn, MA) for both rest and task runs. The first four time points were excluded to allow for signal stabilization. Images were corrected for slice timing and translational and rotational motion by realigning to the first image of the session with INRIAlign (Freire et al., 2002). Images were then normalized to the SPM8 EPI template and resliced to 4 mm for computational efficiency, low pass filtered to exclude frequencies higher than 0.08 Hz, followed by spatial smoothing with 4 mm FWHM. Contributions of motion and physiological noise to the time course of each voxel were removed by including the six motion parameters, signal from ventricle and white matter regions of interest with their respective first temporal derivatives, as regressors of no interest (Wise et al., 2004; Birn et al., 2006; Van Dijk et al., 2010). Further, constant offsets and linear trends were also removed. For the task run, an additional regressor of task conditions was included as being of no interest in order to prevent inflation of functional connectivity estimates by activation differences associated with task conditions (e.g., distracter present vs. absent trials; motor response vs. no motor response). If task conditions are not regressed out, even regions with no moment-to-moment correlations would appear functionally connected because subjects were responding to task conditions over the course of trials [see Jones et al. (2010) for discussion of this point]. Thus, this preprocessing step made the resting and task data comparable, differing only in the subjects' cognitive state [following Gordon et al. (2012a,b)]. The observed pattern of results did not change when task conditions were not regressed out (See Supplementary Materials).

To further restrict the effect of motion on functional connectivity estimates, volumes with greater than 0.5 mm framewise displacement (FD) or temporal derivative of timecourses-root mean square variance over voxel (DVARS) greater than.5% of the whole brain mode value were excluded (as recommended by Power et al. (2012). This “scrubbing” procedure retained 120 timepoints (4 min) for each child for further analysis. For retained volumes, mean FD did not differ between control (Rest: *M* = 0.158 mm, *SD* = 0.061 mm; Task: *M* = 0.167 mm, *SD* = 0.090 mm) and ASD (Rest: *M* = 0.171 mm, *SD* = 0.071 mm; Task: *M* = 0.151 mm, *SD* = 0.069 mm) children during rest (*p* = 0.58) or task (*p* = 0.57); further main effect of state (*p* = 0.63) and the group × state interaction was not significant (*p* = 0.16) indicating that head micromovements did not depend on state. Further, the effects of any residual micromovements were removed by including Mean FD as a regressor in the second-level group analysis [following Saklofske et al. (2012)].

### Local and distant connectivity strength

Following Sepulcre et al. (2010), the resulting smoothed images were used to map the local and distant functional connectivity. The time course of each voxel within a whole-brain mask excluding the cerebellum was correlated to every other voxel's time course, resulting in an *n* × *n* correlation matrix, where n is the dimension of the whole-brain mask (*n* = 33839). The correlation calculation is based on Pearson correlation coefficients (*r*) and thresholded at *p* = 0.001 FDR corrected at the individual level, to exclude less reliable pairwise connections [following Buckner et al. (2009)], resulting in a r threshold range of 0.32–0.34 across individuals, after retaining only positive correlations. For each subject, a resting and task functional connectivity map was computed by averaging the r-to-Z Fisher transformed correlation values, for each voxel to voxels inside (for local connectivity map) and outside (for distant connectivity map) of a 14 mm radius. A 14 mm radius was chosen following Sepulcre et al. (2010) as they observed stable estimates of local connectivity for neighborhood radius values greater than 10 mm and no significant effect on distant connectivity estimates for radius more than 10–14 mm. For discussion of the effects of neighborhood threshold, mask, smoothing kernels and r threshold see Buckner et al. (2009) and Sepulcre et al. (2010). We used connectivity degree weighted by strength (taking both the count of how many links connected to one voxel and their connectivity strength into account– see formulae in Supplementary Materials) as our connectivity estimate rather than connectivity degree alone as used by Sepulcre et al. (2010).

In order to identify regions where group differences in connectivity depended on cognitive state, we tested for Group (ASD, Control) X State (rest, task) interaction in second-level analysis. Subject-specific local and distant functional connectivity maps were entered into separate ANOVA models in SPM8 with Group and State as categorical variables and age and Mean FD as covariates of no interest. This analysis was thresholded at *p* < 0.05 corrected for multiple comparisons based on Monte Carlo simulation (Ward, 2000), which established the correction threshold at height *p* < 0.001, *k* = 5 voxels (for voxel size of 64 mm^3^). For clusters that survived the threshold, functional connectivity values were extracted using MarsBaR toolbox (Brett et al., 2002) from both resting and task runs and graphed to identify the nature of Group and State differences. Further, in regions showing Group × State interaction, we examined whether the magnitude of state-related functional connectivity change was related to inattention. For this analysis, a difference score was computed by subtracting the functional connectivity values from the Resting and Task runs and these difference scores were correlated with the inattention scores from the ADHD Rating Scale, separately for ASD and control children.

**Figure 1:**
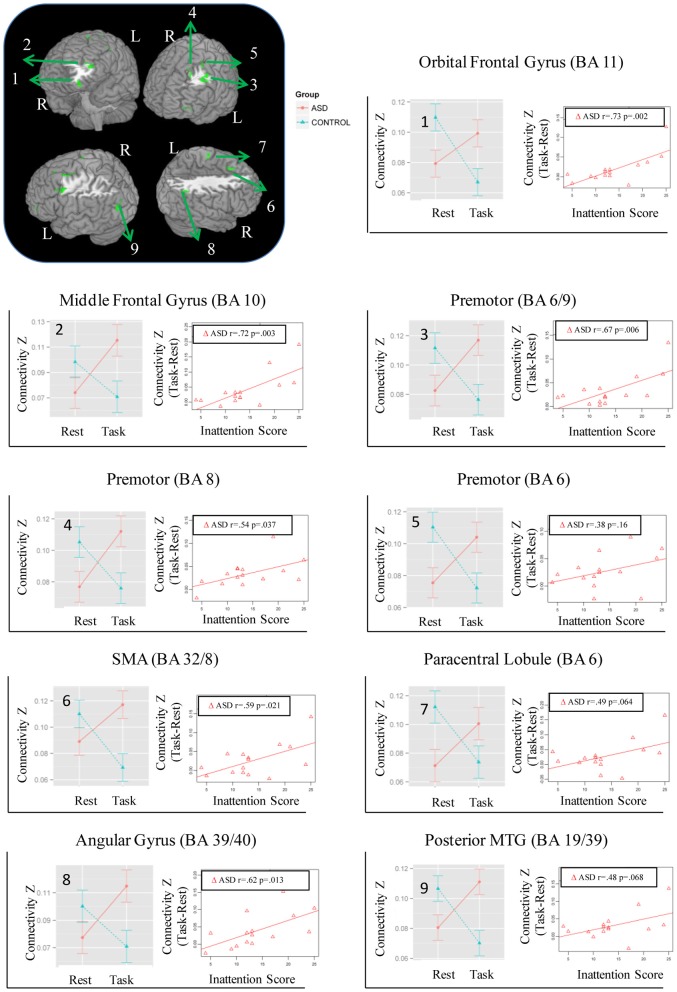
**Regions showing Group × State interaction for distant connectivity.** Each region is identified with a number on the brain image in the top left corner. The corresponding graphs showing the interaction and correlation with inattention scores in the ASD group are identified with the same number.

**Figure 2:**
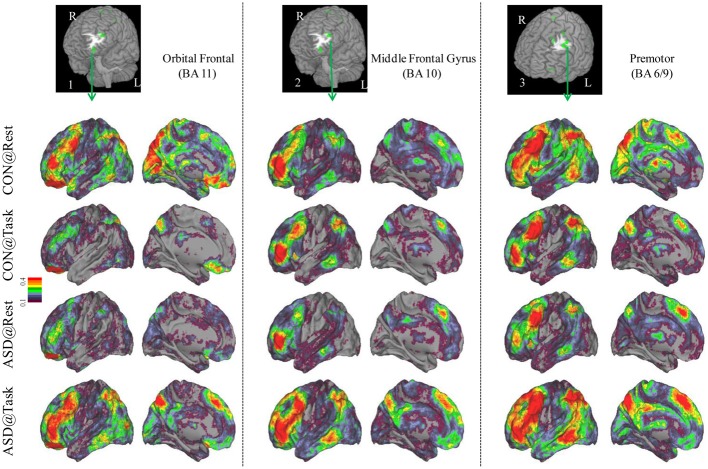
**Seed-based connectivity maps of distant functional connectivity patterns in resting and task states, for three clusters showing Group × Task interaction: left orbital frontal gyrus (BA 11) (left panel), left middle frontal gyrus (BA 10) (middle panel) and left premotor (BA 6/9) (right panel).** Region numbers 1–3 on the left corner in the brain image correspond to the region number in Figure 1.

**Figure 3:**
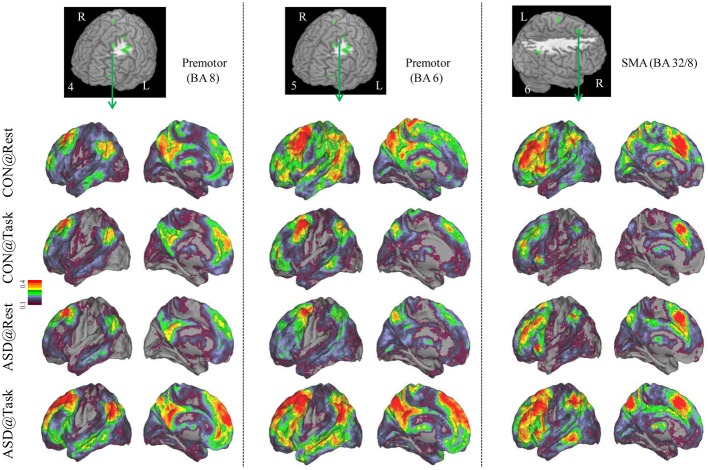
**Seed-based connectivity maps of distant functional connectivity patterns in resting and task states, for three clusters showing Group × Task interaction: left premotor (BA 8) (left panel), left premotor (BA 6) (middle panel) and SMA (BA 32/8) (right panel).** Region numbers 4–6 on the left corner in the brain image correspond to the region number in Figure 1.

**Figure 4:**
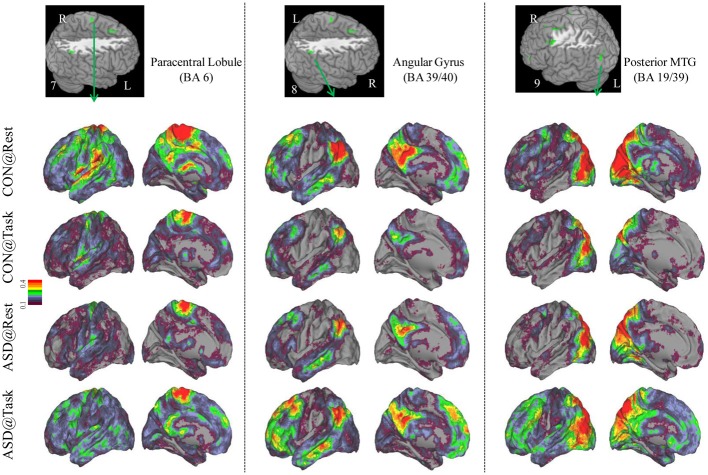
**Seed-based connectivity maps to understand distant functional connectivity patterns in resting and task states, for three clusters showing Group × Task interaction: paracentral Lobule (BA 6) (left panel), right Angular Gyrus (BA 39/40) (middle panel) and Posterior MTG (BA 19/39) (right panel).** Region numbers 7–9 on the left corner in the brain image correspond to the region number in Figure 1.

To visualize the change in distant functional connectivity patterns from resting to task states, we conducted a seed-based connectivity analysis using regions showing Group × Task interaction as seeds. For each subject at each state, the average timecourse of each significant seed cluster was extracted using MarsBaR and correlated with the timecourse of all other voxels in the brain; r values were converted to *Z* using Fisher's transformation. During the correlation calculation, we also regressed out signals of no interest, including timecourses from ventricle, white matter and six motion parameters with their respective first temporal derivatives. Then an averaged group map for each state was generated and visualized (at a range of thresholds 0.1–0.4) on the cortical surface using the population-average, landmark- and surface-based (PALS) surface and plotted using Caret software (Van Essen, 2005). These results are depicted in Figures 1–4. This analysis allowed us to see the nature of change in the pattern of distant connectivity across states.

### Global graph theory measures

We calculated two measures of network topology on a voxel-level graph, global efficiency and modularity, using the brain connectivity toolbox created by Sporns and colleagues (https://sites.google.com/site/bctnet/measures/list); the images were down-sampled to 6 mm voxel size for computational efficiency [see Rubinov and Sporns (2010) and formulae in Supplementary Materials]. These graph measures were calculated by generating the undirected binary whole brain graph (excluding cerebellum as mentioned before), through thresholding the 9736 × 9736 correlation matrix (each 6 mm^3^ voxel to every other voxel) with the same FDR-corrected r threshold used for calculating local and distant connectivity. We also examined the effect of lower *r* thresholds (0.2, 0.1 respectively) on the two graph measures (see Supplementary Materials) to show that our findings were not biased by more stringent *r* threshold selection. For each subject, global efficiency and modularity were calculated for both the resting and task runs and entered into separate ANOVA models in R (http://cran.r-project.org) with Group and State as categorical variables with age and mean FD as covariates of no interest similar to the local/distant connectivity analysis above. Similarly, we also examined whether the magnitude of state-related change in global efficiency and modularity (Task—Resting difference) correlated with the inattention score of the ADHD Rating Scale, separately in the two groups.

## Results

### Behavior

For the task run, groups did not differ in target hits [ASD: *M* = 96.2%, *SD* = 8.0%; Controls: *M* = 100%, *SD* = 0%, *t*_(14)_ = 1.9, *p* = 0.08] and false alarms [ASD: *M* = 0.08%, *SD* = 0.3%; Controls: *M* = 0.2%, *SD* = 0.5%, *t*_(25.7)_ = 1, *p* = 0.33]. However, target response was slower in ASD than control children [ASD: *M* = 602.1 ms, *SD* = 77.6 ms; Controls: *M* = 513.2 ms, *SD* = 70.6 ms, *t*_(28.3)_ = 3.3, *p* = 0.002]. Mean ADHD Rating scores for Inattention [*t*_(21.7)_ = 5.4, *p* < 0.0001] and Hyperactivity-impulsivity [*t*_(21.4)_ = 3.9, *p* < 0.001] were higher in ASD than control children, indicating worse attentional function in ASD (see Table 1).

### Local and distant functional connectivity

While no regions showed a significant Group × State interaction for local connectivity, left frontal, right parietal, and left posterior temporal cortices showed the interaction in distant connectivity (Figure 1). In left frontal cortex, there were six clusters, a medial one including dorsal anterior cingulate extending into Supplementary Motor Area (SMA) (BA 32/8), and five lateral ones including dorsolateral prefrontal (middle frontal gyrus, BA 10; orbital gyrus, BA 11), and three in premotor cortex (BA 8, 6, 6/9). In right parietal cortex, there were two clusters, a dorsomedial one in paracentral lobule (BA 6) and an inferior lateral one near angular gyrus (BA 39/40). The final posterior cluster was in left posterior middle temporal gyrus (BA 19/39). As can be seen in graphs in Figure 1 (cluster information in Table 2), in each of these regions, distant connectivity estimates were reduced in control children but increased in ASD children from resting to task state (See Table S1 for summary of mean, standard deviation and *p*-values in Supplementary Materials). Upon repeating the same analysis without regressing out trial conditions from the task run, similar Group × State interaction regions were found as above but with three exceptions—the paracentral lobule and BA 6 clusters did not survive the corrected threshold and the BA 6/9 cluster became larger (19 voxel vs. 16 voxel) (see Table S2 for summary of mean, standard deviation and p values in Supplementary Materials).

**Table 2:** **Regions showing Group (ASD, Control) × State (Resting, Sustained attention task) interaction for distant functional connectivity**.

**Region**		**MNI coordinates**			**Cluster size (mm^3^)**	**Peak *Z*-score**
		***x***	***y***	***z***		
Left	Orbital frontal gyrus (BA 11)	−18	44	−22	320	3.72
Left	Middle frontal gyrus (BA 10)	−42	48	6	384	3.82
Left	Premotor (BA 6/9)	−42	12	38	1024	3.70
Left	Premotor (BA 8)	−26	20	50	512	4.26
Left	Premotor (BA 6)	−38	4	58	384	4.36
Medial	SMA (BA 32/8)	−2	24	46	384	3.57
Medial	Paracentral lobule (BA 6)	−2	−20	70	320	3.34
Right	Angular gyrus (BA 39/40)	50	−60	38	320	4.66
Left	Posterior MTG (BA 19/39)	−46	−80	18	512	3.87

Seed-based connectivity maps for each of these regions showed that the connectivity map was more focal (i.e., smaller areas in the red-yellow intensity range) during the task relative to the resting run, for the control group. In contrast, for the ASD group, the connectivity map was more diffuse (i.e., larger areas in the red-orange intensity range) during the task relative to the resting run (See Figures 2–4); Figures showing difference maps (*t*-test *p* < 0.005, 5 voxels) comparing groups at each state (Figures S1–S3) and states for each group (Figures S4–S6) are in Supplementary Materials.

### Global graph theory measures

**Figure 5:**
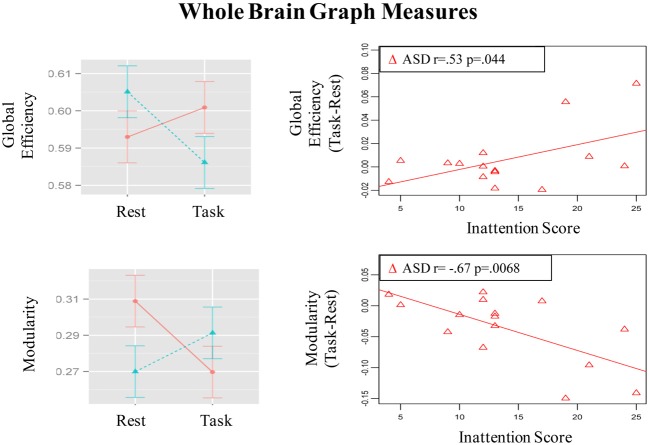
**Graphs depicting Group x State interaction for graph theory measures and correlation of the magnitude of state-related change (Task-Rest) with inattention scores in the ASD group**.

Group × State interaction was observed in global efficiency [*F*_(1, 29)_= 7.78, *p* =.009]; *post-hoc t*-tests showed that global efficiency decreased from resting to the task run in control children [*t*_(15)_ = 2.72, *p* = 0.016] but did not change significantly in ASD children [*t*_(14)_ = 0.96, *p* = 0.36] (See bar graph in Figure 5). Further, the groups did not differ significantly in global efficiency during the resting [*t*_(22, 7)_ = 1.3, *p* = 0.21] or task [*t*_(26, 7)_ = 1. 22, *p* = 0.23] runs.

Modularity also showed a Group × State interaction [*F*_(1, 29)_= 9.45, *p* = 0.005]; *post-hoc t*-tests showed that modularity decreased in ASD children [*t*_(14)_ = 2.62, *p* = 0.02] but did not change significantly in control children [*t*_(15)_ = 1.5, *p* = 0.15] (See bar graph in Figure 5). Further, ASD children had higher modularity than controls [*t*_(23.5)_ = 2.31, *p* = 0.03] during the resting run, but the groups did not differ during the task run [*t*_(28.8)_ = 0.85, *p* = 0.40]. These observed patterns did not change when task conditions were not regressed out (See Table S3).

### Correlation of state-related change in distant connectivity with inattention scores

The magnitude of increase in distant connectivity from resting to the task state in clusters showing Group × State interaction correlated positively with the inattention scores in ASD children, indicating that those with greater attention problems in everyday life showed a stronger increase in distant connectivity from resting to the task run (see scatterplots in Figure 1). Specifically, correlation was significant in dorsolateral prefrontal (BA 11: *r* = 0.73, *p* = 0.002; BA 10: *r* = 0.72, *p* = 0.003), premotor (BA 8: *r* = 0.54, *p* = 0.037; BA 6/9: *r* = 0.67, *p* = 0.006), supplementary motor (BA 32/8: *r* = 0.59, *p* = 0.021), and in right angular gyrus (BA 39/40: *r* = 0.62, *p* = 0.013). In the remaining three clusters, premotor (BA 6, *r* = 0.38, *p* = 0.16), paracentral lobule (*r* = 0.49, *p* = 0.064), and middle temporal (*r* = 0.48, *p* = 0.068), the correlation did not reach significance. The amount of task-related increase of global efficiency (*r* = 0.53, *p* = 0.044) and decrease of modularity (*r* = −0.67, *p* = 0.007) in ASD children also correlated with inattention scores (see scatterplots in Figure 5). Correlations were not significant in control children (*p*s > 0.077), for either regions showing Group × State interaction or graph theory measures.

## Discussion

We used a voxel-wise method to characterize local and distant functional connectivity in two cognitive states, resting and sustained attention, in pre-adolescent children with ASD and control children. Results showed that state-related changes in distant functional connectivity differed between groups in prefrontal, premotor, parietal, and posterior temporal cortical regions known to be associated with cognitive control and spatial attention. In these regions, distant connectivity, defined by the weighted strength of each voxel's temporal correlation with all voxels in the brain outside of its local neighborhood, increased in ASD children but reduced in control children, during sustained attention relative to a preceding resting state. Seed-based maps further confirmed that as hypothesized, reduced distant connectivity in control children reflected a more focal network topology during task than during the resting state. In contrast, contrary to our hypothesis, ASD children showed increased distant connectivity, reflected in a more diffuse network topology, during task than during the resting state. The magnitude of state-related increase in distant connectivity of prefrontal, premotor, and lateral parietal regions correlated positively with ASD children's inattention as measured by parent report on the ADHD Rating Scale. The resting versus task state comparison represents a distinction between attention that is unconstrained relative to that which is constrained by task-goals (e.g., monitoring for a target shape), respectively. As ASD children transition between the unconstrained state to a sustained attention demand, functional connectivity of frontal and parietal regions becomes more widespread, a property that may be maladaptive as it predicted greater attention problems in everyday life.

Some methodological considerations are important to note for interpreting the observed results. First, distant connectivity maps represent moderately high positive correlations (~0.33) between voxels. Further, global signal regression was not performed and therefore, positive/negative correlation value distributions were not altered during preprocessing (Murphy et al., 2009). Thus, interpretation of the observed results is limited to state-related changes in positive functional connectivity. Second, head motion was addressed using “scrubbing” procedures recommended by Power et al. (2012), resulting in retaining 4 min of data in each run for each child. While longer durations are desirable, 4 min is adequate to yield reliable connectivity estimates (Van Dijk et al., 2010). Residual motion was further addressed by using mean FD as a regressor in second-level analysis. As the number of volumes removed and mean FD did not differ between groups, the observed results cannot be attributed to differences in head motion. Third, the sustained attention task included manipulation of distracting information. As our primary aim was to examine effects of cognitive state, task conditions were regressed out, in order to ensure that group differences in connectivity were not driven by differential response to distraction. Importantly, repeating the analysis without regressing out task conditions resulted in a similar pattern of state-related group differences (Table S2), suggesting that the observed group differences were not driven by manipulation of task conditions. Fourth, scan order was fixed, with the resting state run acquired immediately before the task run. Order was not counterbalanced because pre-task and post-task resting state is not identical as task-related functional connectivity persists into the subsequent resting state, suggestive of a cognitive aftereffect (Gordon et al., 2012a). Fifth, our sample sizes of 15/16 children per group are relatively small due to our design requiring two back-to-back fMRI runs satisfying strict motion criteria from the same child. Nonetheless, it is important to note that the small samples limit the generalizability of the observed results.

Distant but not local functional connectivity was sensitive to group differences in modulation by cognitive state. Efficient cortical processing is posited to reflect the balance of connectivity within local regions supported by U-fibers, and across disparate regions supported by long-range white matter tracts (Mesulam, 1998; Schmahmann et al., 2008). While both types of connectivity are present throughout cortex, regions differ in their dominant (e.g. local or distant) connectivity properties. Local hierarchical connections are more representative of sensory cortical areas whereas association cortices such as prefrontal, parietal, lateral temporal, and limbic/paralimbic, have more long-range distributed connections (Felleman and Van Essen, 1991; Mesulam, 1998). In a study with healthy adults, Sepulcre et al. (2010) showed that the local/distant processing topology was paralleled in voxel-wise functional connectivity of low-frequency BOLD signals such that visual and somatosensory cortices showed higher local connectivity whereas association cortices showed higher distant connectivity. Further, while performing a semantic classification task, local and distant connectivity patterns of regions relevant to that task changed relative to a resting state. Here, we found that any state-related changes in local connectivity did not differ between ASD and control children, at least at a threshold that corrected for multiple comparisons. The size of the local neighborhood, 14 mm sphere, was selected based upon Sepulcre et al.'s (2010) recommendation as being optimal for distinguishing regional topography. While that recommendation is based upon adult brain size, it applies to children of the ages examined here as normalization of pediatric brain images to adult stereotactic space has been validated in children as young as 7 years (Burgund et al., 2002; Kang et al., 2003). Lack of significant group differences in state-related modulation of local connectivity suggests that local processing as reflected in voxel-wise BOLD temporal correlations is typical in ASD, at least in the context of transitioning to a relatively easy sustained attention task state.

Distant connectivity was modulated atypically in ASD children during sustained attention relative to a resting state, specifically in regions associated with attentional function. These regions included left dorsolateral prefrontal cortex (BA 10, 11), dorsal anterior cingulate extending to SMA (BA 32/8), and lateral premotor regions (BA 6, 8, 6/9), which are often engaged during tasks requiring cognitive control (Bunge et al., 2002; Vaidya et al., 2005). In addition, there were two parietal clusters, in right paracentral lobule, perhaps associated with motor responses and right inferior parietal cortex associated with spatial attention (Shulman et al., 2010). Finally, there was a cluster in left posterior middle temporal cortex (BA 19/39), a region that children sometimes engage during cognitive control tasks (Rubia et al., 1999; Durston et al., 2003; Vaidya et al., 2005). In all these regions, distant connectivity during sustained attention reduced in control children but increased in ASD children, relative to a resting state. Seed-based connectivity of each of these regions disambiguated the rest-to-task connectivity changes by showing that control children had a focal or less extensive pattern of anterior-posterior connectivity networks during the task relative to resting state. In contrast, ASD children showed the opposite pattern, diffuse or more extensive connectivity networks during the task relative to resting state, suggestive of a lack of selective engagement of task-relevant networks. Such a failure ought to lead to worse performance, which was evident in slower target detection speed in ASD children, while maintaining high accuracy. Further, the extent of increased distant connectivity from rest-to-task states in cognitive control (e.g., prefrontal, medial frontal, premotor) and spatial attention (e.g., lateral parietal) regions was associated with attention problems in everyday behavior as ASD children with larger increases in connectivity had worse inattention scores on the ADHD Rating Scale. Diffuse network engagement during an attentionally demanding state in ASD children may relate to the putative imbalance of inhibitory to excitatory connections associated with glutamatergic (Bejjani et al., 2012) and/or GABAergic dysfuntion (Rojas et al., 2013). If indeed so, then our results suggest that the inhibitory/excitatory milieu of the brain in ASD is modulated by cognitive state in a manner that differs from typical development. Whatever the physiological basis, it appears that in transitioning from a resting to sustained attention state, ASD children exhibited indiscriminate cortical network engagement, which may underlie their functional impairment in the domain of attention.

Group differences in state-related distant connectivity changes were apparent in two graph theory metrics, modularity and global efficiency, which quantify properties of global network organization (Rubinov and Sporns, 2010). Modularity describes the extent to which a network is organized into densely connected modules that are segregated from each other and global efficiency describes the average number of connections to be crossed to go from each voxel to every other voxel in the brain. In control children, global efficiency reduced during sustained attention compared to a resting state; this reduction reflects increased path length, which is consistent with a less extensive network observed during task relative to the resting state. This metric did not show significant difference across states in ASD children. ASD children's modularity reduced during task relative to the resting state, a pattern suggesting increased noise between modules (Bullmore and Sporns, 2009; Rubinov and Sporns, 2010), which is consistent with the observation of a more extensively connected network in ASD children during task than resting state. Even though state-related change was significant only for modularity in ASD children, their amount of change in both graph theory measures predicted inattention scores. Further, comparison of the groups during the resting state showed results that were consistent with past studies using scalp-based imaging measures showing higher modularity [electroencephalography (Barttfeld et al., 2011; Boersma et al., 2013)] in ASD compared to control subjects. While lower global efficiency [magnetoencephalography (Tsiaras et al., 2011)] has been reported in ASD children, it did not differ significantly between groups in the present study. These findings add to the growing volume of studies showing that graph theory metrics are sensitive to inter-individual differences [e.g., age, neurological, and psychiatric disorder (Bullmore and Sporns, 2009)] as well as intra-individual differences [e.g., learning (Bassett et al., 2011), working memory performance (Stevens et al., 2012), IQ (Van den Heuvel et al., 2009)]. Establishing the sensitivity of such whole-brain network metrics to subject factors or cognitive state is an important step in assessing their potential for serving as biomarkers for psychiatric and developmental disorders.

The present findings contribute to developing theories of functional connectivity in ASD in four novel ways. First, they extend the notion that functional connectivity is abnormal in ASD to include transitions across cognitive states. Studies examining functional connectivity during task states that are highly demanding of attention (e.g., theory of mind, working memory, face processing) show reduced connectivity of task-selective networks comprising distant frontal-posterior regions in ASD (Just et al., 2012; Khan et al., 2013). It is plausible that a failure of task-selective engagement such as that suggested by more extensive voxel-wise distant connectivity networks observed here is paralleled in reduced functional connectivity of specific networks or regions. We are unable to effectively test this prediction within the present data because procedures for addressing head motion required excluding volumes with high head motion, making for sparse sampling of individual trial-types.

Second, our findings highlight that examination of highly comorbid deficits in ASD such as attentional function may be insightful about pathophysiology of ASD. Attentional dysfunction is a common comorbid condition in ASD, with over 40% of ASD children estimated to also meet criteria for attention deficit hyperactivity disorder (ADHD) (Leyfer et al., 2006; Yerys et al., 2009b; Sikora et al., 2012). We cannot formally diagnose ADHD in the present sample based solely on parental report on the ADHD Rating scale. However, average scores for inattention and hyperactivity/impulsivity were higher in ASD than control children and 6 of the 15 ASD children had clinically elevated scores for either Inattention or Hyperactivity/impulsivity, consistent with past reports (Yerys et al., 2009b; Rosenthal et al., 2013; Smithson et al., 2013). Attentional and executive dysfunction are common targets for intervention in ASD as they are associated with worse adaptive functioning (Gilotty et al., 2002; Sikora et al., 2012) and outcome in adulthood [see review Hume et al. (2009)]. To the extent that some level of attentional dysfunction always accompanies ASD, it is important to characterize the underlying neural signatures, especially if they prove to be unique to ASD. Thus, it would be important to conduct a similar study in children with ADHD to specify the extent to which our results reflect a general or disorder-specific correlate of transitioning between attentional states.

Third, our graph theory findings contribute to the growing body of studies of large-scale network structure of the brain by showing that modularity and global efficiency were sensitive to ASD and to manipulation of cognitive state. Demonstrating such sensitivity contributes to the potential of such connectivity metrics to serve as biomarkers for psychiatric and developmental disorders. Fourth, the present results highlight the importance of considering cognitive state in current theories of functional connectivity in ASD. It is likely that neither under- nor over-connectivity may characterize ASD in absolute terms but that the nature of alteration may depend upon the specific cognitive state. Mixed findings across task-evoked functional connectivity studies may reflect nuanced differences in the subjects cognitive state induced not just by experimental demands but also the individual's experience of the task as high/low arousing, easy/hard, boring/enjoyable. Furthermore, specific networks may be more susceptible to cognitive state differences than others. As this area of investigation evolves, consideration of task demands, networks, and individual subject characteristics ought to be productive in resolving the status of connectivity abnormality in ASD.

### Conflict of interest statement

The authors declare that the research was conducted in the absence of any commercial or financial relationships that could be construed as a potential conflict of interest.

## Supplementary material

FIGURE S1 **Group differences in seed-based connectivity maps in resting and task states, for three clusters showing Group × Task interaction: left orbital frontal gyrus (BA 11) (left panel), left middle frontal gyrus (BA 10) (middle panel) and left premotor (BA 6/9) (right panel).** Region numbers 1–3 on the left corner in the brain image correspond to the region number in Figure 1.

FIGURE S2 **Group differences in seed-based connectivity maps in resting and task states, for three clusters showing Group × Task interaction: left premotor (BA 8) (left panel), left premotor (BA 6) (middle panel) and SMA (BA 32/8) (right panel).** Region numbers 4–6 on the left corner in the brain image correspond to the region number in Figure 1.

FIGURE S3 **Group differences in seed-based connectivity maps in resting and task states, for three clusters showing Group × Task interaction: Paracentral Lobule (BA 6) (left panel), right Angular Gyrus (BA 39/40) (middle panel) and Posterior MTG (BA 19/39) (right panel).** Region numbers 7–9 on the left corner in the brain image correspond to the region number in Figure 1.

FIGURE S4 **State differences in seed-based connectivity maps within each group, for three clusters showing Group × Task interaction: left orbital frontal gyrus (BA 11) (left panel), left middle frontal gyrus (BA 10) (middle panel) and left premotor (BA 6/9) (right panel).** Region numbers 1–3 on the left corner in the brain image correspond to the region number in Figure 1.

FIGURE S5 **State differences in seed-based connectivity maps within each group, for three clusters showing Group × Task interaction: left premotor (BA 8) (left panel), left premotor (BA 6) (middle panel) and SMA (BA 32/8) (right panel).** Region numbers 4–6 on the left corner in the brain image correspond to the region number in Figure 1.

FIGURE S6 **State differences in seed-based connectivity maps within each group, for three clusters showing Group × Task interaction: Paracentral Lobule (BA 6) (left panel), right Angular Gyrus (BA 39/40) (middle panel) and Posterior MTG (BA 19/39) (right panel).** Region numbers 7–9 on the left corner in the brain image correspond to the region number in Figure 1.
