# Supplementary material for: Atypical modulation of distant functional connectivity by cognitive state in children with Autism Spectrum Disorders
Source: Front Hum Neurosci. 2013 Aug 27;7:482. doi: 10.3389/fnhum.2013.00482 (PMC3753572; doi:10.3389/fnhum.2013.00482)
Supplement: FIGURE S1 — Group differences in seed-based connectivity maps in resting and task states, for three clusters showing Group × Task interaction: left orbital frontal gyrus (BA 11) (left panel), left middle frontal gyrus (BA 10) (middle panel) and left premotor (BA 6/9) (right panel). Region numbers 1–3 on the left corner in the brain image correspond to the region number in Figure 1. [file Presentation1.PDF]

Supplementary Materials
Captions for Supplementary Figures

Figure S1. Group differences in seed-based connectivity maps in resting and task states, for three clusters showing Group X Task interaction: left orbital frontal gyrus (BA 11) (left panel), left middle frontal gyrus (BA 10) (middle panel) and left premotor (BA 6/9) (right panel). Region numbers 1-3 on the left corner in the brain image correspond to the region number in Figure 1.

Figure S2. Group differences in seed-based connectivity maps in resting and task states, for three clusters showing Group X Task interaction: left premotor (BA 8) (left panel), left premotor (BA 6) (middle panel) and SMA (BA 32/8) (right panel). Region numbers 4-6 on the left corner in the brain image correspond to the region number in Figure 1.

Figure S3. Group differences in seed-based connectivity maps in resting and task states, for three clusters showing Group X Task interaction: Paracentral Lobule (BA 6) (left panel), right Angular Gyrus (BA 39/40) (middle panel) and Posterior MTG (BA 19/39) (right panel). Region numbers 7-9 on the left corner in the brain image correspond to the region number in Figure 1.

Figure S4. State differences in seed-based connectivity maps within each group, for three clusters showing Group X Task interaction: left orbital frontal gyrus (BA 11) (left panel), left middle frontal gyrus (BA 10) (middle panel) and left premotor (BA 6/9) (right panel). Region numbers 1-3 on the left corner in the brain image correspond to the region number in Figure 1.

Figure S5. State differences in seed-based connectivity maps within each group, for three clusters showing Group X Task interaction: left premotor (BA 8) (left panel), left premotor (BA 6) (middle panel) and SMA (BA 32/8) (right panel). Region numbers 4-6 on the left

corner in the brain image correspond to the region number in Figure 1.

Figure S6. State differences in seed-based connectivity maps within each group, for three clusters showing Group X Task interaction: Paracentral Lobule (BA 6) (left panel), right Angular Gyrus (BA 39/40) (middle panel) and Posterior MTG (BA 19/39) (right panel). Region numbers 7-9 on the left corner in the brain image correspond to the region number in Figure 1.

List of Formulae

The following formulae were used to calculate the functional connectivity measures, including regional measures, local and distant connectivity strength, and graph-theory measures, global efficiency and modularity. Global efficiency and modularity were calculated using the brain connectivity toolbox created by Sporns and colleagues (https://sites.google.com/site/bctnet/measures/list). We calculated connectivity strength as a modified version of connectivity degree used by Sepulcre et al. (2010) where connectivity degree was weighted by strength, taking both the count of how many links connected to one voxel and their correlation value into account. Our distant connectivity strength measure is highly linked to the basic degree measure -- which provides a link between our regional measures and graph measures.

1) Local Connectivity Strength C_{ij} for a voxel (node) i

$$C_i = \sum_{j=1}^{N} c_{i\ j} \Big/ N , \quad i \neq j \tag{1}$$

where j is the index of the voxel inside the neighborhood above the threshold r, N denotes the total number of voxels inside the neighborhood, c_{ij} denotes the connectivity strength (r to Z transformed) between voxel i and j.

2) Distant Connectivity Strength C_{ij} for a voxel (node) i

$$C_i = \sum_{j=1}^{N} c_{ij} \Big/ N \quad , \quad i \neq j \tag{2}$$

where j is the index of the voxel outside the neighborhood above the threshold r, N denotes the total number of voxels inside the neighborhood, c_{ij} denotes the connectivity strength (r to Z transformed) between voxel i and j.

3) Global Efficiency (Latora and Marchiori, 2001)

$$E = \frac{1}{n}\sum_{i \in N}^{N} E_i = \frac{1}{n}\sum_{i \in N}^{N} \frac{\sum_{j \in N, j \neq i} s_{ij}^{-1}}{n-1} = \frac{1}{n}\sum_{i \in N}^{N} \frac{\sum_{j \in N, j \neq i} \left(\sum_{a_{uv} \in g_{i \leftrightarrow j}} d_{uv}\right)^{-1}}{n-1} \tag{3}$$

Where N is the set of all nodes in the network, n is the number of nodes, s_{ij} is the shortest path length(distance) between node d_{uv} denotes the connection status between voxel i and j (either 1 for connected pair or 0 for unconnected pair).

4) Modularity (Newman, 2006)

$$E = \frac{1}{l}\sum_{i, j \in N} \left(d_{ij} - \frac{\sum_{j \in N} d_i \sum_{i \in N} d_j}{l}\right)\delta_{m_i, m_j} \tag{4}$$

where m_i is the module containing node i , and δ_{m_i, m_j} =1 if $m_i = m_j$, and 0 otherwise, d_{ij} denotes the connection status between voxel i and j (either 1 for connected pair or 0 for unconnected pair), l is the number of links.

Group X Sate Interaction Details

In the manuscript, we tested for Group (ASD, Control) X State (rest, task) interaction in second-level analysis for local and distant connectivity measures, during which subject-specific local and distant functional connectivity maps were entered into separate ANOVA models in SPM8. We identified 9 clusters that showed this interaction pattern for distant connectivity, as seen in Figure 1 and Table 2. We then extracted the distant connectivity at both task and resting states for each cluster for all the subjects. In Table S1 below, we provide the mean and standard deviation of the distant connectivity at each state and each group, as well as the post hoc t test results to assess the interaction pattern. We also included the p value of correlation for magnitude of functional connectivity change from resting to task runs (Task-Resting difference) with inattention scores on the ADHD Rating Scale, separately in the two groups. From Table S1, we can see that all clusters showed significant group differences at rest (Control>ASD) and at task (ASD>Control), and also state-related changes within each group**.**

We also provide a similar table (Table S2) for the same analysis but without the regression of task condition in the task run. Note similar group x state interaction patterns were found for the previously reported clusters with same coordinates except that the paracentral lobule and premotor region (BA6) did not survive the corrected threshold.

As in Figure 2-4, we used the seed-based network measure to visualize the entire topology that relates to the specific clusters showing group X state interaction, so that the reader can see how the topology associated with those specific clusters changes by state for each group. We have then conducted two-sample t-tests at each state for each map and find that results confirm what is apparent on visual inspection - at rest, controls show greater connectivity than ASD children for every map and at task, ASD children show greater connectivity than controls (Figures S1-S3). There are no regions that show the

opposite, i.e., ASD > controls at rest or Controls > ASD at task. We have also conducted paired t-tests in each group between rest and task states for each map and find that results confirm that from rest to task state, controls show decreased connectivity (more focal as we suggested) while ASD children show increased connectivity (more diffuse) for every map (Figures S4-S6). There are no regions that show the opposite, i.e., Task > Rest for control group or Rest > Task for ASD group. We have rendered the results at p < .005, 5 voxels.

Effect of r threshold for Graph Theory Measures

In order to examine whether the results from graph theory measures are sensitive to r threshold, we performed similar group x state interaction for global efficiency and modularity on two lower thresholds (r =0.2 and 0.1) graphs. We found the interaction remained significant and the pattern of results the same as that reported for the more stringent r threshold in the main manuscript. The amount of task-related increase of global efficiency and decrease of modularity in ASD children also correlated with inattention scores. Statistical results details see Table S3.

Table S1. Detailed summary of distant functional connectivity strength for clusters showing Group X State interaction.

Regions	Mean(SD) at Rest Control	Mean(SD) at Task Control	Mean(SD) at Rest ASD	Mean(SD) at Task ASD	p Between Group at Rest	p Between group at task	p Between States Control	p Between States ASD	p GroupX State	p correlation with Inattention CON	p correlation with Inattention ASD
Orbital Frontal (BA 11)	0.11(.04)	0.068(.02)	0.08(.018)	0.098(.039)	0.014	0.014	8.00E-05	0.066	2.0E-05	0.2	0.002
Middle Frontal (BA 10)	0.10(.045)	0.072(.023)	0.075(.022)	0.11(.058)	0.086	0.017	0.015	0.017	0.0004	0.122	0.003
Premotor (BA 6/9)	0.11(.044)	0.077(.027)	0.083(.026)	0.12(.052)	0.039	0.018	0.005	0.002	4.0E-05	0.49	0.006
Premotor (BA 8)	0.11(.047)	0.076(.026)	0.077(.019)	0.11(.033)	0.041	0.004	0.017	0.0005	5.0E-05	0.338	0.037
Premotor (BA 6)	0.11(.044)	0.073(.032)	0.076(.018)	0.1(.035)	0.011	0.019	0.002	0.005	1.0E-05	0.699	0.16
SMA(BA 32/8)	0.11(.042)	0.070(.029)	0.09(.023)	0.12(.051)	0.1	0.006	0.0005	0.03	5.0E-05	0.077	0.021
Paracentral Lobule (BA 6)	0.11(.043)	0.074(.032)	0.071(.022)	0.099(.051)	0.003	0.116	0.0004	0.05	0.0001	0.841	0.064
Angular Gyrus (BA 39/40)	0.1(.032)	0.07(.031)	0.077(.024)	0.12(.051)	0.025	0.008	0.013	0.009	0.0003	0.817	0.013
Posterior MTG (BA 19/39)	0.11(.04)	0.07(.029)	0.08(.021)	0.11(.044)	0.028	0.006	5.00E-05	0.009	4.0E-06	0.307	0.068

Table S2. Detailed summary of distant functional connectivity strength for clusters showing Group X State interaction without regressing out task conditions.

Regions	Mean(SD) at Rest Control	Mean(SD) at Task Control	Mean(SD) at Rest ASD	Mean(SD) at Task ASD	p Between Group At Rest	p Between group at task	p Between States Control	p Between States ASD	p GroupX State	p correlation with Inattention CON	p correlation with Inattention ASD
Orbital Frontal (BA 11)	0.11(.040)	0.068(.020)	0.080(.018)	0.099(.041)	0.014	0.015	8.1E-05	0.067	2.5E-05	0.196	0.002
Middle Frontal (BA 10)	0.098(.045)	0.072(.023)	0.075(.022)	0.114(.058)	0.086	0.017	0.016	0.016	0.0004	0.118	0.003
Premotor (BA 8)	0.105(.047)	0.077(.026)	0.077(.019)	0.111(.034)	0.041	0.004	0.018	0.0006	0.00005	0.309	0.032
Premotor (BA 6/9)	0.109(.042)	0.075(.027)	0.082(.026)	0.114(.053)	0.04	0.019	0.004	0.003	4E-05	0.438	0.008
SMA(BA 32/8)	0.11(.042)	0.070(.029)	0.090(.023)	0.116(.052)	0.108	0.006	0.0006	0.034	6.7E-05	0.076	0.022
Posterior MTG (BA 19/39)	0.106(.040)	0.070(.030)	0.080(.021)	0.111(.046)	0.027	0.007	3.8E-05	0.011	5.8E-06	0.272	0.099
Angular Gyrus (BA 39/40)	0.1(.032)	0.071(.031)	0.077(.024)	0.115(.052)	0.025	0.008	0.013	0.009	0.0003	0.801	0.013

Table S3. Detailed summary of global efficiency and modularity at correlation(r) values .02 and .01. The rows labeled FDR correspond to results included in the main manuscript.

Measures	Mean(SD) at Rest Control	Mean(SD) at Task Control	Mean(SD) at Rest ASD	Mean(SD) at Task ASD	p Between Group At Rest	p Between Group at task	p Between States Control	p Between States ASD	p Group X State	p correlation with Inattention CON	p correlation with Inattention ASD
Global_Efficiency_FDR	0.605(.031)	0.587(.026)	0.594(.016)	0.6(.033)	0.207	0.232	0.016	0.355	0.009	0.375	0.044
Modularity_FDR	0.27(.057)	0.29(.0624)	0.308(.031)	0.271(.064)	0.03	0.401	0.154	0.02	0.005	0.617	0.007
Global_Efficiency_02	0.678(.032)	0.663(.028)	0.665(.018)	0.674(.033)	0.16	0.305	0.037	0.154	0.008	0.313	0.041
Modularity_02	0.21(.053)	0.226(.054)	0.242(.032)	0.211(.050)	0.052	0.432	0.222	0.013	0.007	0.655	0.008
Global_Efficiency_01	0.74(.032)	0.727(.029)	0.726(.020)	0.736(.030)	0.135	0.401	0.065	0.086	0.008	0.282	0.044
Modularity_01	0.166(.047)	0.179(.048)	0.192(.029)	0.165(.039)	0.076	0.405	0.293	0.006	0.009	0.656	0.01

Figure S1

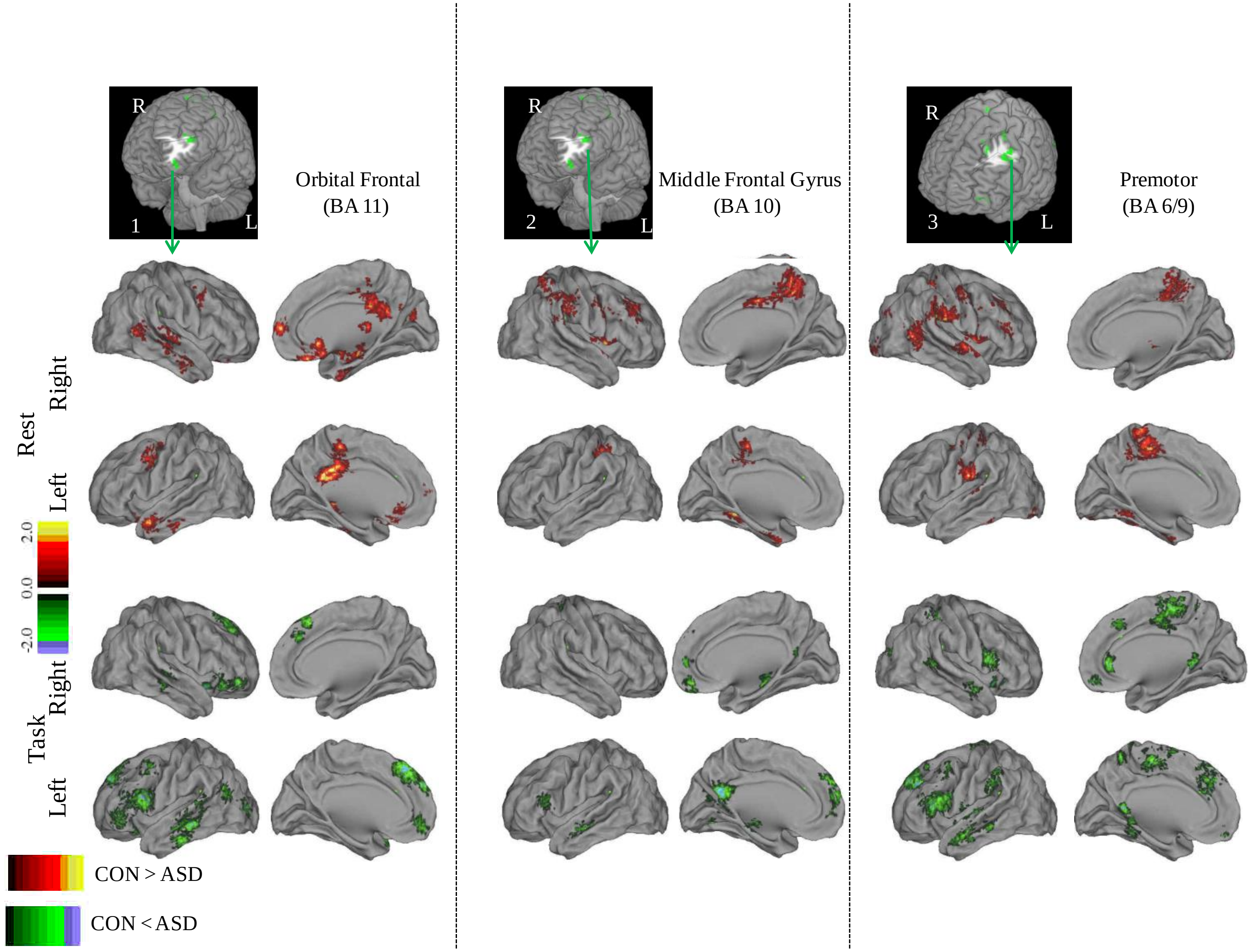
R
1
L
Orbital Frontal
(BA 11)
R
2
L
Middle Frontal Gyrus
(BA 10)
R
3
L
Premotor
(BA 6/9)
Rest
Right
Left
2.0
0.0
-2.0
Task
Right
Left
CON > ASD
CON < ASD

Figure S2

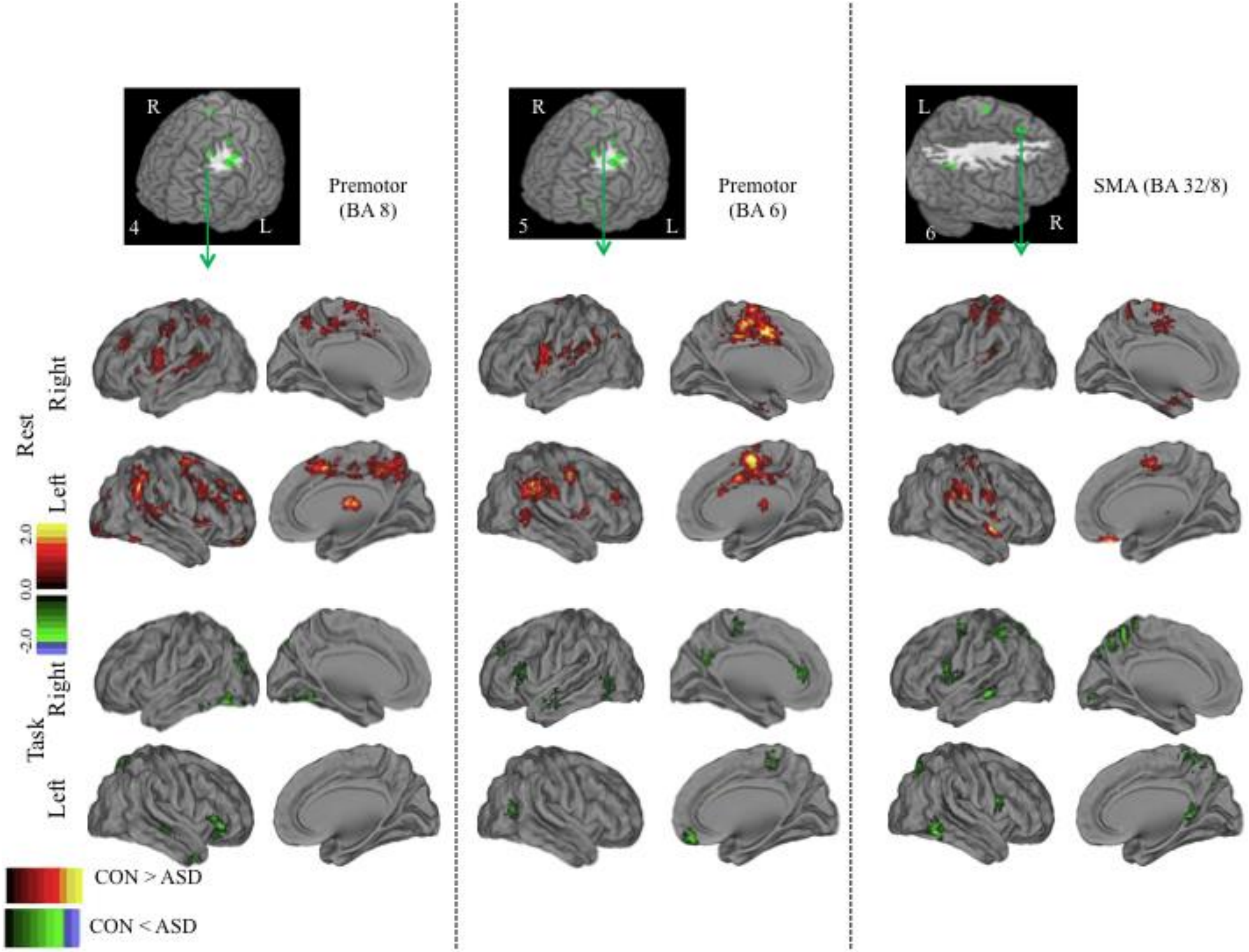

R
4
L
Premotor
(BA 8)
R
5
L
Premotor
(BA 6)
L
6
R
SMA (BA 32/8)
Rest
Right
Left
Task
Right
Left
2.0
0.0
-2.0
CON > ASD
CON < ASD

Figure S3

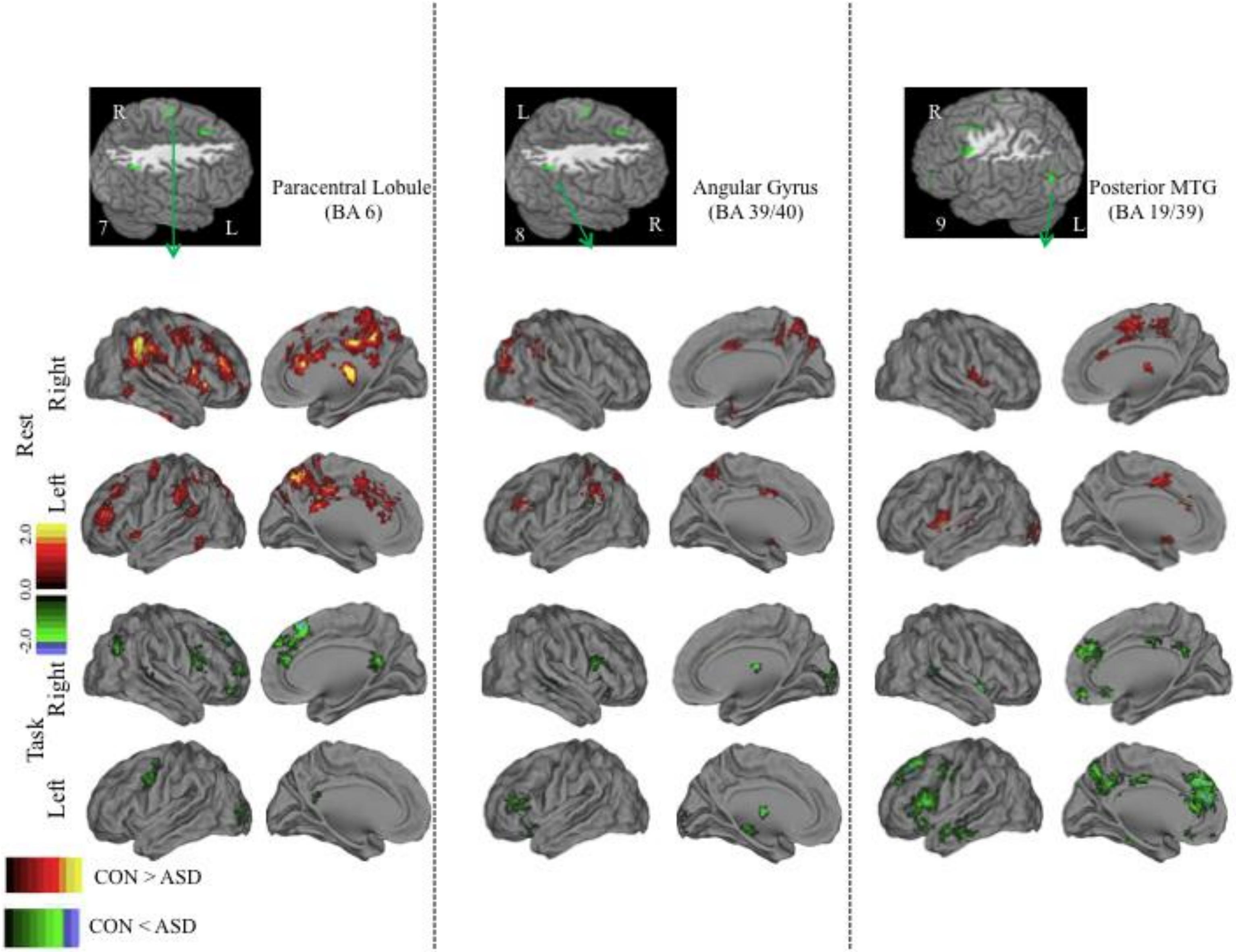
R
L
7
Paracentral Lobule
(BA 6)
L
R
8
Angular Gyrus
(BA 39/40)
R
L
9
Posterior MTG
(BA 19/39)
Rest
Right
Left
Task
Right
Left
2.0
0.0
0.0
-2.0
CON > ASD
CON < ASD

Figure S4

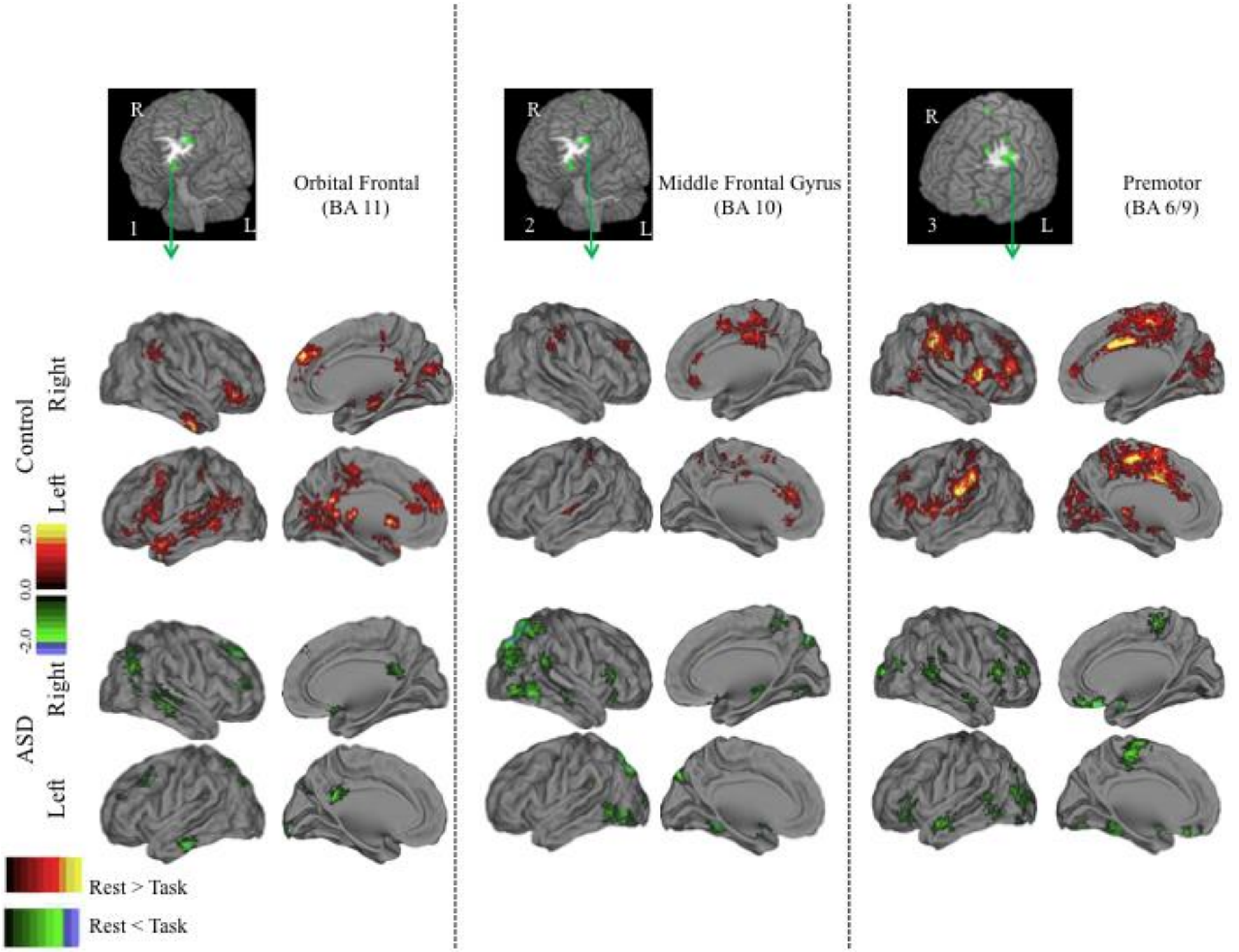
R
1
L
Orbital Frontal
(BA 11)
R
2
L
Middle Frontal Gyrus
(BA 10)
R
3
L
Premotor
(BA 6/9)
Control
Right
Left
ASD
Right
Left
2.0
0.0
-2.0
Rest > Task
Rest < Task

Figure S5

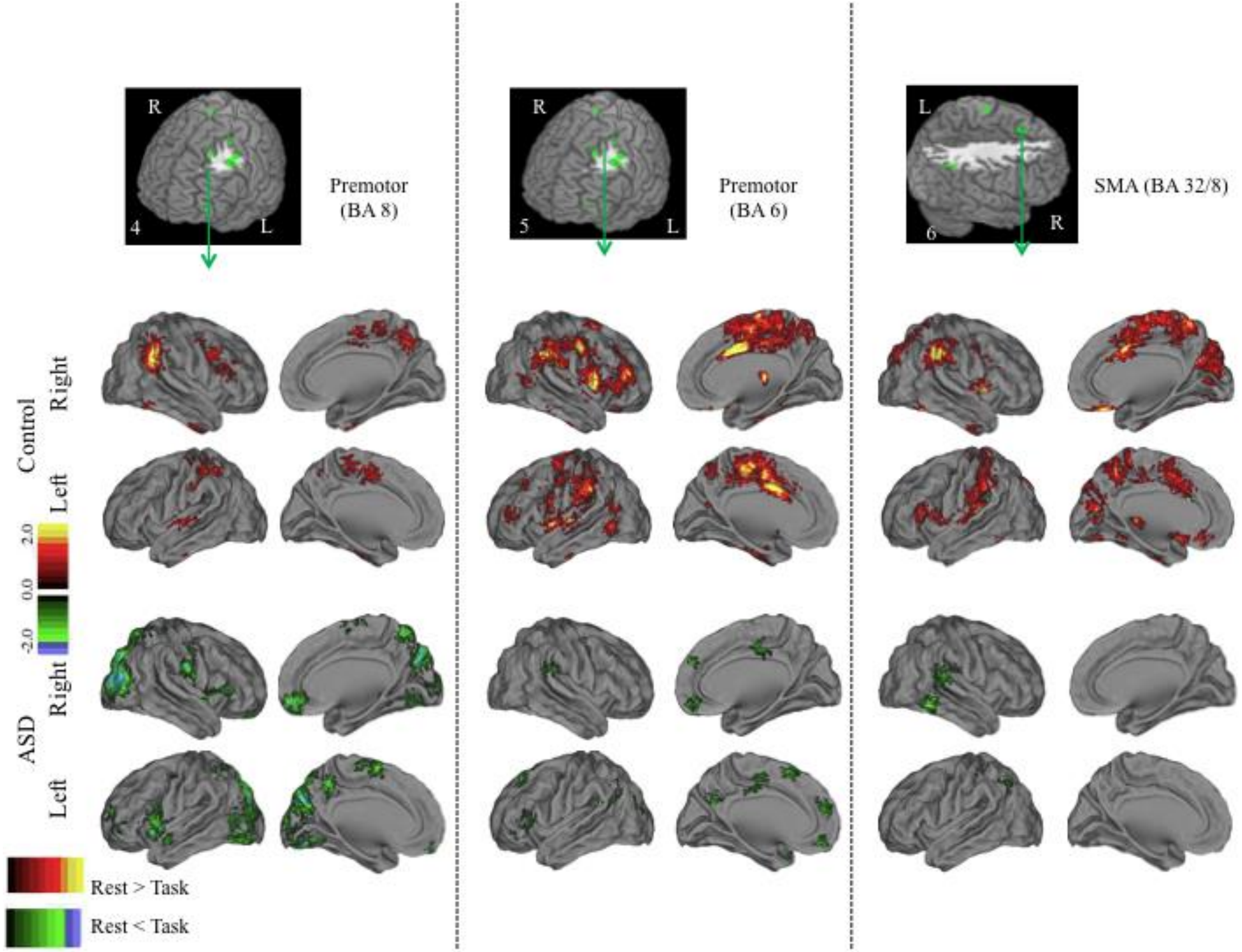
R
4
L
Premotor
(BA 8)
R
5
L
Premotor
(BA 6)
L
6
R
SMA (BA 32/8)
Control
Right
Left
2.0
0.0
-2.0
ASD
Right
Left
Rest > Task
Rest < Task

Figure S6

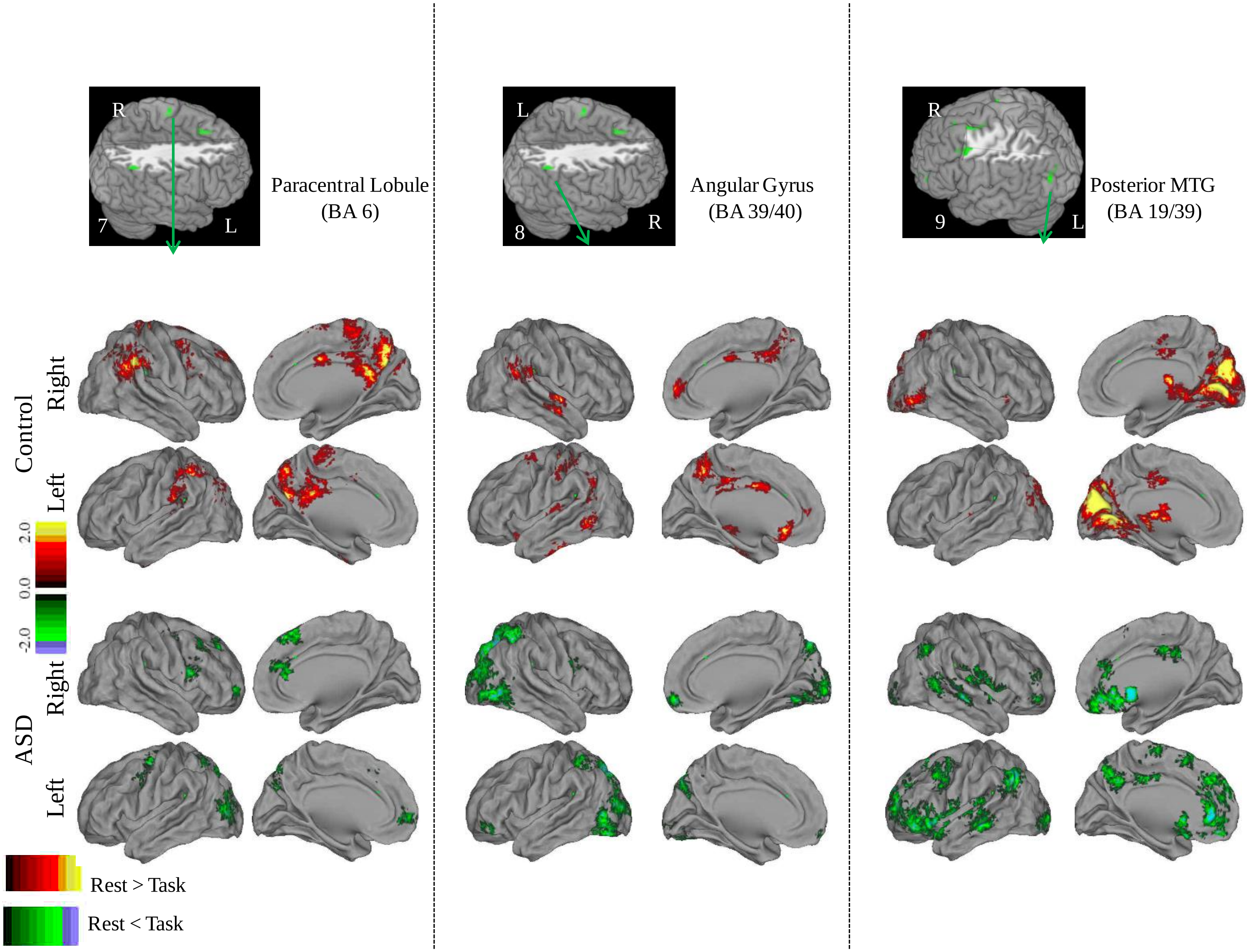
R
7
L
Paracentral Lobule
(BA 6)
L
8
R
Angular Gyrus
(BA 39/40)
R
9
L
Posterior MTG
(BA 19/39)
Control
Right
Left
ASD
Right
Left
2.0
0.0
-2.0
Rest > Task
Rest < Task
